# Analysis of Retinal Peripapillary Segmentation in Early Alzheimer's Disease Patients

**DOI:** 10.1155/2015/636548

**Published:** 2015-10-18

**Authors:** Elena Salobrar-Garcia, Irene Hoyas, Mercedes Leal, Rosa de Hoz, Blanca Rojas, Ana I. Ramirez, Juan J. Salazar, Raquel Yubero, Pedro Gil, Alberto Triviño, José M. Ramirez

**Affiliations:** ^1^Instituto de Investigaciones Oftalmológicas Ramón Castroviejo, Universidad Complutense de Madrid (UCM), 28040 Madrid, Spain; ^2^Departamento de Oftalmología y ORL, Facultad de Medicina, UCM, 28040 Madrid, Spain; ^3^Departamento de Oftalmología y ORL, Facultad de Óptica y Optometría UCM, 28040 Madrid, Spain; ^4^Servicio de Geriatría, Hospital Universitario Clínico San Carlos, 28040 Madrid, Spain

## Abstract

Decreased thickness of the retinal nerve fiber layer (RNFL) may reflect retinal neuronal-ganglion cell death. A decrease in the RNFL has been demonstrated in Alzheimer's disease (AD) in addition to aging by optical coherence tomography (OCT). Twenty-three mild-AD patients and 28 age-matched control subjects with mean Mini-Mental State Examination 23.3 and 28.2, respectively, with no ocular disease or systemic disorders affecting vision, were considered for study. OCT peripapillary and macular segmentation thickness were examined in the right eye of each patient. Compared to controls, eyes of patients with mild-AD patients showed no statistical difference in peripapillary RNFL thickness (*P* > 0.05); however, sectors 2, 3, 4, 8, 9, and 11 of the papilla showed thinning, while in sectors 1, 5, 6, 7, and 10 there was thickening. Total macular volume and RNFL thickness of the fovea in all four inner quadrants and in the outer temporal quadrants proved to be significantly decreased (*P* < 0.01). Despite the fact that peripapillary RNFL thickness did not statistically differ in comparison to control eyes, the increase in peripapillary thickness in our mild-AD patients could correspond to an early neurodegeneration stage and may entail the existence of an inflammatory process that could lead to progressive peripapillary fiber damage.

## 1. Introduction

Alzheimer's disease (AD), the most common cause of dementia, afflicts 67 of every 1000 persons over age 65. Its prevalence and incidence increase exponentially with age [1, 2]. In 2006, the worldwide prevalence of Alzheimer's was 26.6 million, and by 2050, the prevalence will quadruple, meaning that by that time 1 in 85 persons worldwide will be living with the disease [2].

AD is characterized by a decline in cognitive function, loss of learning and memory, and the formation of neuritic plaques and neurofibrillary tangles, primarily in the cerebral cortex [3, 4].

The retina is a projection of the brain, and a number of similarities between AD pathology and several distinct retinal degenerations have been described [5, 6]. The retinal nerve fiber layer (RNFL) is composed of retinal-ganglion cell axons that form the optic nerve. Decreased thickness of the RNFL can reflect retinal neuronal-ganglion cell death and axonal loss in the optic nerve [7, 8].

The RNFL reportedly thins with aging [9, 10]. Some studies have also shown a decrease of the RNFL in AD in addition to aging [7, 8, 11–16]. Hinton et al. [17] were the first to show histopathological evidence of retinal-ganglion cell loss and optic-nerve degeneration in patients with AD. These findings were then confirmed in several follow-up studies [18–21]. Indeed, the large magnocellular cell axon degeneration in AD has been documented [19, 22]. Other histopathology studies [23–28], however, have failed to confirm these findings and suggest that methodological differences were responsible for the contradictory results, due to a different postmortem delay in axon count or difficulties in obtaining well-preserved myelinated axons.

Currently it is thought that retinal ganglion cell (RGC) loss in AD might result from amyloid deposits in the eye and/or retina. Amyloid-beta plaques as well as oligomers have been reported in postmortem retinal tissue from patients with AD and in a mouse model of AD, as well as in human retinal tissue* in vivo* [29]. Therefore, amyloid accumulation in the eye or retina of patients with AD may result in the degeneration of RGC in parallel to amyloid-beta-related neurodegeneration in the brain [29].

Diagnosis and progression of AD, especially early cases, are complicated because of imprecise neuropsychological testing, sophisticated but expensive neuroimaging techniques, and invasive sampling of cerebrospinal fluid [30, 31]. Improved methods for screening and early detection are essential to identify cognitively normal individuals who have a high risk of developing AD, so that treatment can be developed to delay the progression of the disease [32]. Currently, there is no definitive antemortem diagnosis for AD, and new biomarkers for diagnosis are therefore needed. Over the last few decades, very accurate tools for analyzing the eye fundus have been developed (i.e., OCT, laser polarimetry), opening new ways of examining the retina* in vivo.*

OCT is a reliable noninvasive technique, routinely used in ophthalmology to visualize and quantify the layers of the retina. OCT enables quantitative cross-sectional imaging of the RNFL and macular volume. A recent study published by our group [33] has shown that in mild-AD patients the first affected area of the retina is the macular area. As the neurodegeneration progresses, a significant decline in peripapillary RNFL thickness will become apparent.

The goal of the present study was to examine in detail peripapillary and macular segmentation in order to determine which is the earliest thinned area in patients with mild AD which may be used, in the future, as a predictive tool.

## 2. Material and Methods

### 2.1. Subjects

To select patients, we reviewed the Database of the Memory Unit of the Hospital Clinico San Carlos in Madrid (Spain), consisting of a total of 2635 patients. First, we excluded the patients with a Global Deterioration Scale (GDS) over 4 and then those with a mood or psychiatric disorder. Next, we took into account 87 patients with mild AD. These patients, according to the National Institute of Neurological and Communicative Disorders and Stroke-AD and Related Disorders Association and the Diagnostic and Statistical Manual of Mental Disorders IV, had mild cognitive impairment according to the Clinical Dementia Rating scale. Then ophthalmic medical records of these patients were reviewed, excluding patients who were previously diagnosed with an ophthalmological pathology (glaucoma or suspected glaucoma, media opacity, and retinal diseases). After this analysis, 29 patients with AD satisfied all the requirements to participate in the study (GDS over 4 and free of ocular disease and systemic disorders affecting vision in their medical record). Of the 29 mild-AD patients and 37 age-matched control subjects selected (normal MMSE scores), 6 mild-AD patients and 9 age-matched control subjects were subsequently excluded due to posterior pole pathology including macular degeneration, drusen, suspicion of glaucoma, glaucoma, epiretinal membrane, or cataract that prevented ocular examination. Because of this selection, 23 patients with mild AD and 28 age-matched control subjects were considered for the study. Informed consent was obtained from both groups. The research followed the tenets of the Declaration of Helsinki, and the protocol was approved by the local ethics committee.

### 2.2. Methods

For the ophthalmological part of the study, the right eye of each patient was analyzed. All participants met the following inclusion criteria: being free of ocular disease, AREDS Clinical Lens Standards <2, retinal drusen, and systemic disorders affecting vision; having a best corrected VA of 20/40; having a ±5 spherocylindrical refractive error; and having intraocular pressure of less than 20 mmHg. For screening, all AD patients and control subjects underwent a complete ophthalmologic examination, including assessment of VA, refraction, anterior segment biomicroscopy, applanation tonometry (Perkins MKII tonometer, Haag Streit-Reliance Medical, Switzerland), dilated fundus examination, and OCT. The RNFL thickness and macular thickness were measured by OCT Model 3D OCT-1000 (Topcon, Japan) after pupil dilatation. The RNFL thickness was scanned 3 consecutive times per patient in each area studied. The mean values were considered for statistical analysis. All tests were performed by the same optometrist (ESG) under the same conditions. These tests were selected considering that in this developmental stage of the disease the results were not influenced by the patient's cognitive impairment.

**Figure 1:**
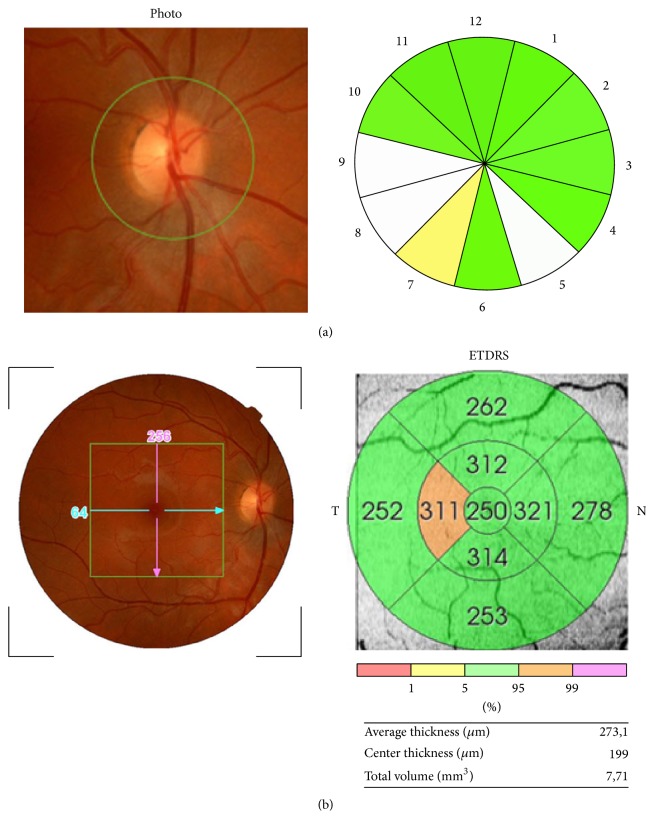
OCT report of retinal nerve fiber layer (RNFL) thickness analysis. (a) Peripapillary OCT. The thickness for each 12-o'clock hour position with the 3-o'clock position as nasal, 6-o'clock position as inferior, 9-o'clock position as temporal, and 12-o'clock position as superior was evaluated. (b) Macular OCT. Diagram showing the concentric rings and quadrants considered for analysis of the macular RNFL thickness and measurements automatically provided by the analyzer.

The peripapillary RNFL thickness parameters evaluated in this study were average thickness (360° measurement), thickness for each 12-o'clock hour position with the 3-o'clock position as nasal, 6-o'clock position as inferior, 9-o'clock position as temporal, and 12-o'clock position as superior. Macular RNFL thickness data were displayed in three concentric rings centered in the foveola that were distributed as follows: a central macular ring, 1 mm away from the fovea; an inner macular ring, 3 mm away from the fovea; and an outer macular ring, 6 mm away from the fovea. As a result, the total area studied made up a 6 mm macular map. In addition, the inner and outer rings were each divided into four quadrants (superior, inferior, nasal, and temporal) (Figure 1). The total volume of the macula as provided by the OCT was also calculated. The good scan criteria were determined as the signal-to-noise ratio >30 and accepted A-scans >95% in fast RNFL scanning. All measurements are given in microns, according to the calibration provided by the manufacturers and the total volume in mm^3^.

### 2.3. Statistical Analysis

The data are reported as mean values ± SD. The differences between mild AD and control eyes were analyzed using the Mann-Whitney test. Data for the statistical analysis were introduced and processed in a SPSS 19.0 (SPSS Inc©, Inc, Chicago, IL, USA). A *P* value of <0.05 was considered statistically significant.

## 3. Results

Demographic and clinical data for the mild-AD patients and control group are shown in Table 1. No statistically significant differences in age, gender, or educational level were found between the study groups. The MMSE scores in mild-AD patients were significantly decreased in comparison with age-matched control subjects (Table 1). All mild-AD patients had MMSE values higher than 17.

### 3.1. Optical Coherence Tomography

**Figure 2:**
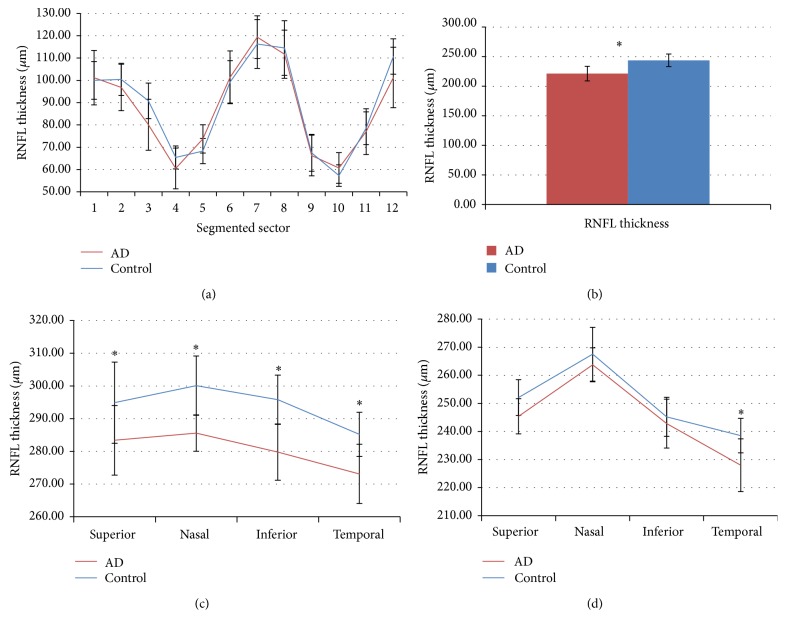
Mean data of RNFL thickness against eye quadrants assessed with optical coherence tomography (OCT). (a) Peripapillary segmentation retinal nerve fiber layer, (b) Central macular ring (1 mm away from the fovea). (c) Inner macular ring (3 mm away from the fovea). (d) Outer macular ring (6 mm away from the fovea). ^*∗*^ *P* value < 0.01.

*Peripapillary RNFL Segmentation Thickness*. Peripapillary RNFL thickness values (Figure 2(a)) showed no statistical difference between mild-AD patients and control subjects (Table 2).

Although the differences were not significant in any of the sectors, it was shown that peripapillary sectors 2, 3, 4, 8, 9, 11, and 12 were thinner in the mild-AD patients than in controls; in peripapillary sectors 1, 5, 6, 7, and 10 the retina in mild-AD patients was thicker with respect to the control (Figure 2(a); Table 2).

*Macular RNFL Thickness and Total Volume*. As we reported in a previous study [33] the analysis of the RNFL revealed that, in patients with mild AD, the values for the central ring (fovea) (Figure 2(b)) and the four inner quadrants (3 mm from the fovea) (Figure 2(c)) were significantly decreased in comparison with control subject (*P* < 0.05 in both instances; Mann-Whitney *U* test) (Table 2). The RNFL thickness of the outer macular quadrants (6 mm from the fovea) (Figure 2(d)) in patients with mild AD was diminished in comparison with control subjects; however, only the values of the outer temporal quadrant were significantly lower (*P* < 0.05; Mann-Whitney *U* test) (Table 2).

The total macular volume was significantly reduced in mild-AD patients in comparison with control subjects (*P* < 0.05; Mann-Whitney *U* test) (Table 2).

## 4. Discussion 

Alzheimer's dementia syndromes, like all neurodegenerative diseases, lack objective disease- and stage-specific biomarkers [34]. As a part of the CNS, the retina or neural portion of the eye shares many features with the brain, including embryological origin as well as anatomical and physiological characteristics. Its peripheral location provides an accessible and noninvasive way of examining brain pathology [35]. OCT is a reliable noninvasive technique that enables quantitative cross-sectional imaging of the RNFL [36].

Thinning of the RNFL has been found in several neurological diseases, such as Parkinson's disease [16, 37–39], dementia with Lewy Bodies [16], amnestic mild cognitive impairment [8, 15], neuromyelitis optica [40], migraine [41], and AD [8, 11–17, 20, 27, 36, 37, 42–45]. The loss of RNFL thickness in AD is linked to a depletion of retinal-ganglion cells and optic-nerve axons [13, 14, 32, 46, 47]. It has been postulated that the defects in RNFL may be the earliest sign of AD, even prior to damage to the hippocampal region that impairs memory [36]. In addition, published data suggest an association between the thinning of RNFL and severity of AD [8, 11].

In the present work, we compare the peripapillary RNFL segmentation thickness, macular thickness, and the total macular volume in mild-AD patients and age-matched control subjects. One of the relevant issues of the study was that the sample analyzed here was homogeneous in that (i) all patients had recently been diagnosed as having mild AD (GDS 4, Reisberg scale [48]) with mean MMSE score values of 23.7 ± 3.3; (ii) all the individuals were Caucasians; and (iii) there were no significant differences in age or educational level among the groups. The results of our study showed a difference between the peripapillary RNFL segmentation and the macula thickness in our mild-AD patients in that only the macular thickness was significantly decreased in comparison with the control group.

Widespread axonal degeneration in the optic nerve was found in a postmortem study of patients with AD [17]. Morphometric analysis of the whole-mount retina has shown that Alzheimer's patients had a predominant loss of the largest class of retinal-ganglion cells (M-cells), which could be a primary process or a consequence of retrograde neurodegeneration occurring in the cortical regions [19].* In vivo* studies using different methodologies have confirmed optic-nerve-fiber damage in AD when compared with controls. Optic disc pallor, pathologic disc cupping, and thinning of the neuroretinal rim and the RNFL have been reported in studies based on the subjective evaluation of fundus photographs [11, 42] and the optic-nerve analyzer [42].

There is controversy on the reduction of the peripapillary RNFL thickness measured by OCT in AD. A reduction in the thickness of all peripapillary RNFL quadrants as measured by OCT has been reported [12, 43], and it has been suggested that this morphologic abnormality is related to retinal dysfunction as revealed by abnormal patterns in electroretinogram responses [43]. However, some OCT studies on peripapillary thickness in AD [8, 13, 15, 16, 36, 44, 45, 49] found that the RNFL thinning was restricted to the superior quadrant [13, 44, 50–52] or to the superior and inferior quadrants [15, 37, 45] in comparison with control subjects. Some studies have correlated cognitive decline with decreased RNFL thickness [33, 50, 53]. It has been suggested that the inferior quadrant of the RNFL may be a more specific and sensitive area than other RNFL quadrants in predicting the deterioration of cognitive status to reflect retinal abnormality in the early stages of AD [15, 50]. The reason for the variability of the results among studies could be related to MMSE scores. Thus, Parisi et al. [43] and Iseri et al. [12], whose patients had more advanced AD (ranges of MMSE scores 11 to 19 and 8 to 28, resp.), showed a reduction in RNFL thickness in all peripapillary quadrants. Kesler et al. [15], whose patients had a mean MMSE score of 23.6, showed a decrease in the superior and inferior peripapillary quadrants. By contrast, both Berisha et al. [13], whose Alzheimer's patients had higher MMSE scores (17 to 30), and Paquet et al. [8], whose patients had a mean MMSE score of 22.6, found a thickness reduction only in the superior peripapillary quadrant, postulating this finding as being the earliest peripapillary retinal damage in AD patients. In our patients, with MMSE values similar to those reported by Berisha and Paquet, the reduction of mean peripapillary RNFL thickness did not reach statistical significance in comparison to control, but peripapillary RNFL thickness diminished or increased, depending on the segment studied. It should be noted that the thinning sectors of papilla corresponded to 2, 3, 4, 8, 9, and 11, while sectors 1, 5, 6, 7, and 10 showed a thickening. These values differ from those found in the same sectors of the controls but in any case reach statistical significance.

Most authors, although working in more advanced stages of disease (MMSE > 23.7), agree that the peripapillary RNFL thinning is significant in the superior and inferior sectors [13, 37, 44]. However, sectors 1, 5, 6, 7, and 10 in our patients showed thickening. This dissimilarity could be explained because of difference in the stage of the disease, which in our case corresponded to a much earlier stage. This tendency towards greater thickness, although not statistically significant, could be related to the findings of Ascaso et al. in the macula of patients with mild cognitive impairment (MCI) and AD. Patients with MCI had greater RNFL thickness compared to AD and controls, suggesting that this difference could be caused by inflammation after gliosis neuronal death [54]. Similarly, the increase in peripapillary thickness in our mild-AD patients in the sectors 1, 5, 6, 7, and 10, corresponding to the superior and inferior sectors, may indicate a phase of inflammation and gliosis of neural tissue prior to the degenerative process.

Reactive astrogliosis in the brain is a well-known feature of AD, but its role in AD is not well understood. Reactive astrogliosis tends to be focal in AD. Reactive astrocytes are intimately associated with amyloid plaques or diffuse amyloid deposits. Astrocytes surround them with dense layers of processes as if forming miniature scars around them, perhaps to wall them off and act as neuroprotective barriers [55]. It is plausible that, in the early stages of the disease, microglial activation could help remove amyloid plaques, while in later phases proinflammatory cytokines induced by microglia could contribute to neurodegenerative process [56, 57].

In the same way, retinal neurodegenerative diseases are also associated with chronic microglial activation and neuroinflammation. In the degenerating retina, endogenous signals activate microglial cells, leading to their local proliferation, migration, enhanced phagocytosis, and secretion of cytokines, chemokines, and neurotoxins. These immunological responses and the loss of limiting control mechanisms may contribute significantly to retinal tissue damage and proapoptotic events in retinal neurodegeneration [57–59]. A limitation to be considered in our study, as well as those reported in the literature on RNFL thickness evaluation by OCT, is the number of patients included. Studies on early-stage Alzheimer's patients are difficult to perform, one reason being that these patients usually come for diagnosis at advanced stages of the disease. Taking this into consideration and the homogeneity of the patients included in the present work, we consider that our data provide preliminary evidence to warrant a more extensive study.

## 5. Conclusions

In the present study, the analysis of the OCT values of both peripapillary and macular RNFL thickness in patients with mild AD (MMSE = 23.7) showed that only in the macula was there a significant thickness reduction compared to aged-matched controls. Our data, taken together with those reported in the literature, move us to propose the hypothesis that the first affected area of the retina in mild AD is the macular area, where, due to the arrangement of the multilayer bodies of the ganglion cells, the decrease is easier to detect.

Subsequently, as the neurodegeneration progresses, a significant decline in peripapillary RNFL thickness will become apparent. The study of the peripapillary segmentation reveals, in a more accurate way, the changes that occur in RNFL thickness in relation to the macular-thickness changes. In this sense, our patients with mild AD differed with respect to controls, although without reaching statistical significance; perhaps due to the early stage of the disease. In addition, the increase in peripapillary thickness in our mild-AD patients may indicate the existence of an inflammatory process that would lead to neurodegeneration of the peripapillary fibers. More extensive studies should be conducted to test these findings.

## Figures and Tables

**Table 1:** Demographic and clinical data of the study groups.

	AD	Control	*P* value
	(*n* = 23)	(*n* = 28)	
Age^§^	79.3 ± 4.6	72.3 ± 5.1	0.274

Gender			
Male	9	9	0.615
Female	14	19	

Race	Caucasian	Caucasian	

MMSE^§^	23.3 ± 3.1	28.2 ± 1.9	0.001^*∗*^
	Range (17–29)	Range (25–31)	

^§^Mean value ± SD; ^*∗*^ *P* < 0.01 [AD, Alzheimer's disease; MMSE, Mini-Mental State Examination; SD, standard deviation].

**Table 2:** RNFL thickness and total macular volume.

Retinal area of study		AD group^§^	Control group^§^	% RNFL decrease	*P* value
Peripapillary thickness (*μ*m)	Sector 1	101.2 ± 24.4	100.0 ± 16.9	1.24	0.790
	Sector 2	96.8 ± 20.8	100.4 ± 14.4	−3.62	0.618
	Sector 3	80.1 ± 22.9	90.8 ± 16.0	−11.85	0.084
	Sector 4	60.5 ± 18.3	65.4 ± 10.3	−7.60	0.464
	Sector 5	73.7 ± 12.7	68.3 ± 11.3	7.83	0.173
	Sector 6	101.4 ± 23.7	99.3 ± 19.0	2.02	0.790
	Sector 7	119.4 ± 19.1	116.3 ± 21.9	2.62	0.756
	Sector 8	111.7 ± 21.6	114.5 ± 24.5	−2.48	0.564
	Sector 9	66.3 ± 18.2	67.5 ± 16.5	−1.80	0.877
	Sector 10	60.7 ± 13.8	57.3 ± 9.6	5.99	0.464
	Sector 11	77.0 ± 20.6	78.5 ± 14.7	−1.96	0.94
	Sector 12	101.3 ± 27.1	110.7 ± 15.8	−8.45	0.335

Foveal thickness (*μ*m)	Fovea	221.2 ± 21.6	243.7 ± 24.8	−9.24	0.015^*∗*^

Inner macular quadrant (*μ*m)	Superior area	283.4 ± 11.1	294.9 ± 18.1	−3.91	0.002^*∗*^
	Inferior area	279.8 ± 18.1	295.8 ± 13.5	−5.40	0.002^*∗*^
	Nasal area	285.6 ± 17.2	300.1 ± 15.1	−4.83	0.007^*∗*^
	Temporal area	273.1 ± 12.7	285.2 ± 14.6	−4.22	0.002^*∗*^

Outer macular quadrant (*μ*m)	Superior area	245.4 ± 12.5	252.1 ± 13.7	−2.65	0.084
	Inferior area	242.8 ± 17.4	245.2 ± 13.9	−0.99	0.531
	Nasal area	263.7 ± 12.1	267.5 ± 19.1	−1.41	0.110
	Temporal area	228.0 ± 18.8	238.5 ± 12.3	−4.43	0.009^*∗*^

Total macular volume (mm^3^)		7.1 ± 0.3	7.3 ± 0.3	9.34	0.024^*∗*^

^§^Mean value ± SD; ^*∗*^ *P* < 0.05 [AD, Alzheimer's disease; RNFL: retinal nerve fiber layer; SD, standard deviation].
